# Development of Metallo (Calcium/Magnesium) Polyurethane Nanocomposites for Anti-Corrosive Applications

**DOI:** 10.3390/ma15238374

**Published:** 2022-11-24

**Authors:** Manawwer Alam, Mohammad Altaf, Mukhtar Ahmed, Mohammed Rafi Shaik, Rizwan Wahab, Jilani Purusottapatnam Shaik, Mohammad Shahzad Samdani, Ashfaq Ahmad

**Affiliations:** 1 Department of Chemistry, College of Science, King Saud University, P.O. Box 2455, Riyadh 11451, Saudi Arabia; 2 Department of Zoology, College of Science, King Saud University, P.O. Box 2455, Riyadh 11451, Saudi Arabia; 3 Department of Biochemistry, College of Science, King Saud University, P.O. Box 2455, Riyadh 11451, Saudi Arabia; 4 College of Science, King Saud University, P.O. Box 2455, Riyadh 11451, Saudi Arabia

**Keywords:** renewable resource, rapeseed oil, metallo nanocomposite, anti-corrosive

## Abstract

Long-term corrosion protection of metals might be provided by nanocomposite coatings having synergistic qualities. In this perspective, rapeseed oil-based polyurethane (ROPU) and nanocomposites with calcium and magnesium ions were designed. The structure of these nanocomposites was established through Fourier-transform infrared spectroscopy (FT-IR). The morphological studies were carried out using scanning electron microscopy (SEM) as well as transmission electron microscopy (TEM). Their thermal characteristics were studied using thermogravimetric analysis (TGA). Electrochemical experiments were applied for the assessment of the corrosion inhibition performance of these coatings in 3.5 wt. % NaCl solution for 7 days. After completion of the test, the results revealed a very low i_corr_ value of 7.73 × 10^−10^ A cm^−2^, a low corrosion rate of 8.342 × 10^−5^ mpy, impedance 1.0 × 10^7^ Ω cm^2^, and phase angle (approx 90°). These findings demonstrated that nanocomposite coatings outperformed ordinary ROPU and other published methods in terms of anticorrosive activity. The excellent anti-corrosive characteristic of the suggested nanocomposite coatings opens up new possibilities for the creation of advanced high-performance coatings for a variety of metal industries.

## 1. Introduction

Oil crops are the second most important energy source in meals consumed by humans, behind grains. They are also used as food for agricultural animals. These oils are a great source of raw materials for a variety of industrial products [1]. Soy accounts for approximately 57% of global oilseed production. Rapeseed (20%), cotton (11%), and sunflower (8%) take the next three spots [2]. Rapeseed (*Brassica napus*) is a significant plant that provides oil in temperate climate zones whose production is increasing in Europe as well as globally. This plant is grown on over 3 million European hectares, accounting for more than 60% of the region engaged by oilseeds. Rapeseed is a good source of fat (oil). Rapeseed oil (RO) has precious properties and therefore is being used in a variety of industries. RO is highly valued in the food as well as cosmetic industries owing to its high vitamin and unsaturated fatty acid content, taste, and abundance of health benefits. Also, it is utilized as a diesel fuel additive and standalone fuel [3]. Additionally, RO can be transformed into monomers for valuable polymer production like polyols, epoxies, alkyd, and polyurethane.

Polyurethanes (PU) are a category of polymers that are very much elastic in terms of properties, making them useful in all aspects of society and business. With an average expenditure of more than 12 million tonnes, these polymers are among the most prominent plastics. As a result of their excellent mechanical characteristics and great chemical resistance, PUs are replacing conventional materials like ceramics, metals, and rubber in numerous applications [4]. One of the most fascinating areas of modern chemistry study has been the impregnation of metal ions in a polymer matrix. These metallopolymers act as new materials with desirable properties that can be controlled by ligand coordination to the metal center [5]. Moreover, metallo-PU nanocomposites offer a combination of properties that are discrete from their organic and inorganic counterparts and have corrosion applications. Liquid crystals, sensors, conducting, electro-catalysts, light-emitting materials, and high thermal conductivity are other potential applications for metallopolymers [6]. Metallo-PUs are also the most promising candidates for advanced corrosion-protection coatings.

Modern society makes substantial use of metallic substrates in a variety of things, including consumer goods, aviation, and infrastructures [7]. All these materials are prone to various electrochemical reactions causing corrosion, thereby resulting in deterioration. Materials science has long been involved in the study of the protection of metal from corrosion [8]. Among many other techniques to solve this purpose, the application of protective coating and utilization of corrosion inhibitors are considered to be the best as they are cost-effective and convenient [9]. Protective coatings work as a fundamental barrier between the metal and the harsh environment to inhibit the ingress of corrosive ions [10]. These coatings help in protecting metal, improving their functionalities and increasing their lifetime [11,12]. However, after several days of exposure, corrosive species like ions, oxygen, and water tend to penetrate the polymers [13]. As soon as water molecules penetrate through the coating surface, corrosion of metal starts beneath the film, which effectively reduces the adhesion of the coating with the metal surface [14]. Therefore, improving the protection capabilities of the coated system is an important aspect to study. For this reason, various techniques such as enhancement of polymer properties, preparing metallic surfaces, or working on the intermediate layers formed during corrosion have been extensively studied [15]. In order to enhance the properties of polymers and make them more durable, metal ions are incorporated into the polymer matrix. These metal ions form a covalent bond with the available functional groups and help in the formation of a compact structure that does not allow the penetration of harmful species [16]. The substantial improvement of these coating systems can be measured in terms of applied amplitude and alternating potential signal and studied through electrochemical impedance spectroscopy (EIS) technique qualitatively [11,17].

The novelty of this work involves (*i*) the utilization of RO for the synthesis of a hydroxy-terminated prepolymer, which was further transformed into polyurethane with the help of toluene 2,4-diisocyanate (TDI), (*ii*) the incorporation of a divalent metal ion such as Ca^2+^ and Mg^2+^ in RO based polymer matrix to enhance its mechanical as well as anticorrosive properties and lastly (*iii*) the anti-corrosive performance of RO based metallo-PUs were explored for the first time through electrochemical impedance spectroscopy (EIS).

The structure of synthesized PU was established with the help of Fourier transform infrared (FTIR) spectroscopy. Also, pull-off adhesion test and microhardness measurements were used to investigate PU coatings applied on mild steel. PU was chosen as the innovative polymer matrix to explore potential relationships between electrochemical behavior and mechanical characteristics of the metal ion-containing systems due to its excellent adhesion to metallic substrates and good hardness. This investigation proposes a green approach for the synthesis of metallo-polyurethane nanocomposite for advanced anti-corrosive application.

## 2. Materials and Methods

### 2.1. Materials

Rapeseed oil (Loba Chemie, Maharashtra, India), diethanolamine (Loba Chemie, Maharashtra, India), sodium metal, sodium chloride (BDH chemicals Ltd., Poole, England), magnesium acetate (Sigma Aldrich, St. Louis, MO, USA), calcium acetate (Winlab, Limited, Leicestershire, MA, USA), toluene (Fisher scientific company, Branchburg, NJ, USA), toluene 2,4-diisocyanate (TDI, 80%, Acros Organics, Morris Plains, NJ, USA), diethyl ether, methanol (Sigma Aldrich, St. Louis, MO, USA).

### 2.2. Methods

The FTIR spectra are recorded using the NaCl window on Spectrum 100 (Perkin Elmer, Waltham, MA, USA) for functional group analysis. X-ray photoelectron spectroscopy, XPS (JPS-9030; JEOL, Tokyo, Japan). X-ray diffraction XRD (D2 Phase X-ray diffractometer, Bruker, Karlsruhe, Germany) to analyze the nature of polymers. Thermogravimetric analysis (TGA) and differential scanning calorimetry (DSC) (Mettler Toledo AG, Analytical CH-8603, Schwerzenbach, Switzerland) were recorded in an N_2_ environment with a heating rate of 10 °C/min. for thermal stability. Morphological examinations were carried out using SEM-EDS on a Jeol, Japan, JED-2200 Series, and TEM Jeol-1011. The physicomechanical properties of M-ROPU coated mild steel panels were investigated using impact resistance (IS: 101-part 5 sec 3, 1988), scratch hardness (BS 3900), cross-hatch adhesion (ASTM D3359-02), pencil hardness test (ASTM D3363), and conical mandrel bend (ASTM D3281-84). An elcometer coating thickness gauge (Model 456; Elcometer Instruments, Manchester, UK) was used to measure the thickness of the coatings, and a glossmeter was used to verify the gloss (KSJ MG6-F1, KSJ Photoelectrical Instruments Co., Ltd., Quanzhou, China). Contact angle measured with a CAM 200 Attention goniometer, Biolin Scientific, Amsterdam, The Netherlands.

Electrochemical impedance spectroscopy (EIS) and Potentiodynamic polarization (PDP) techniques were used to evaluate the corrosion inhibition performance of M-ROPU-coated samples. The test was performed at ambient temperature; a three-electrode device was used to measure impedance in a corrosive 3.5% NaCl solution. The working electrode was an M-ROPU-coated mild steel sample, the counter-electrode was platinum, and the reference electrode was KCl-filled silver, all of which were connected to an Autolab potentiostat/galvanostat (PGSTAT204-FRA32, Metrohom Autolab B.V. Kanaalweg 29-G, 3256 KM, Utrecht, The Netherlands). The exposed surface area of the M-ROPU coating was set to 1.0 cm^2^ using a PortHoles electrochemical sample mask. The EIS values were recorded in the frequency range of 1–10^5^ Hz, with a 10 mV amplitude of the sinusoidal voltage. PDP values were recorded potential range ± 250 mV with respect to OCP and scan rate 10 mV/s. The impedance data were analyzed and fit using the NOVA 2.1 program.

### 2.3. Synthesis of Rapeseed Oil-Based Diol (R-Diol)

Rapeseed oil-based diol (R-diol) (Chemical name: *N,N bis*(*2-hydroxy ethyl*) rapeseed oil fatty amide) was synthesized according to our previously reported work [18,19].

### 2.4. Synthesis of Ca-R-Diol/Mg-R-Diol

A simple one-pot, two-step in-situ reaction was used to develop metallopolymer nanocomposites producing zero-waste. Ca-R-diol/Mg-R-diol were synthesized in different compositions (Ca/Mg-R-diol-1 to Ca/Mg-R-diol-4) by the reaction of R-diol and various amounts of metal acetate, i.e., 1 wt. %, 3 wt. %, 5 wt. % and 7 wt. % respectively. The optimization of Ca/Mg-R-diol was carried out as per our previously reported research [20]. A three-necked flat-bottom flask with a thermometer, water condenser, and nitrogen intake tube was filled with freshly manufactured R-diol. The assembly was set over a magnetic stirrer in an oil bath that was maintained at a constant temperature of 100 °C. During the first step, R-diol serves as monomer and solvent both. Finely powdered metal salt (Ca/Mg Ac) was added in particular amounts (1, 3, 5, and 7 wt. %) while being continuously stirred over the course of 1 h. After all of the metallic ions had been added, the temperature was increased and kept at 120 °C. Thin layer chromatographic (TLC) technique, FTIR spectra as well as the solubility of the intermediates were periodically recorded at room temperature to confirm the progress and completion of the reaction.

### 2.5. Synthesis of Ca-ROPU/Mg-ROPU

M-R-diol (M = Ca/Mg) was further reacted to obtain rapeseed oil-based polyurethane and named ROPU. The metal ion containing ROPU was named as per the metal used, i.e., Ca-ROPU for ROPU incorporated with calcium and Mg-ROPU for ROPU incorporated with magnesium. The temperature of the reaction mixture was then lowered to 35 °C. Later, TDI was added gradually in optimized percentage, i.e., 35 wt. % within 15 to 20 min, with regard to the metal coordination pre-polymeric units. Xylene (10–20 wt. %) was added at this point to manage the system’s overall viscosity. Once more, the reaction was monitored by TLC and FT-IR spectra.

## 3. Results and Discussion

**Scheme 1:**
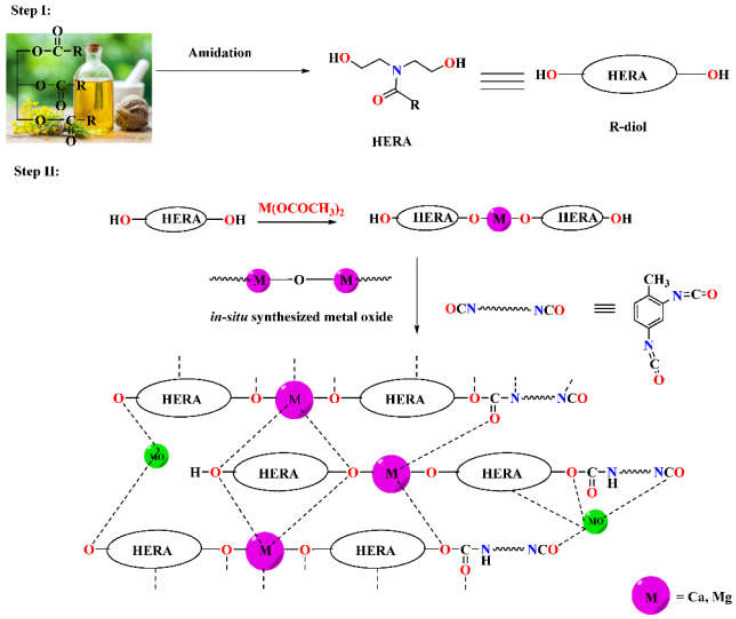
Synthesis of metallo polyurethane from rapeseed oil.

In order to synthesize metallo-ROPU nanocomposites for corrosion protection coatings, a two-step in-situ process was used in this work. Fatty amide derivatives were prepared from RO in the first step, which serves as a reactive electron-donating ligand system for the metal ions. These green ligands were coordinated with the cationic metal centers to produce a 2D or extended 1D network. The system becomes more flexible but has a comparatively greater viscosity due to these increases in chain length and the rise in molecular weight of individual subunits. The reaction was carried out at a temperature of 120 °C, which encourages the process to advance by removing tiny molecules. The development and conclusion of the reaction were led by the FT-IR and TLC plate recording. However, an additional cross-linking agent (TDI) was added to this reaction mixture at room temperature of 30 °C in order to create and stabilize a 3D interpenetrating network. The urethane linkages resulted in a robust 3D polyurethane framework (Scheme 1). The electrostatic interactions, Van der Waals forces, and intermolecular H-bonding, as shown in Scheme 1, were responsible for the geometrical arrangement around the metal ion.

ROPU resin was produced through an addition polymerization reaction between the –OH groups of M-R-diol and the –NCO groups of TDI to form urethane. Whereas, Ca/Mg impregnated ROPU was produced through in-situ oxidative polymerization, resulting in a blended mixture containing both organic and inorganic components within one phase. Furthermore, metallo-ROPU nanocomposites were developed through an in-situ solventless polymerization procedure, as reported in the literature. Also, during the course of the reaction, metal oxides (–M–O–M–) are also produced from the interaction of M-R-diol with free metal acetate.

Additionally, this in-situ technique results in the simultaneous interaction of the hydroxyl group of M-R-diol with the –N=C=O group of TDI and metal ions with the CO group of urethanes, causing the formation of highly cross-linked metallo-ROPU nanocomposites. By residing in the interstitial sites of the polymeric backbone, these metal ions induce cementing mechanisms, which leads to the development of a more compact structure.

### 3.1. Spectral Studies

**Figure 1:**
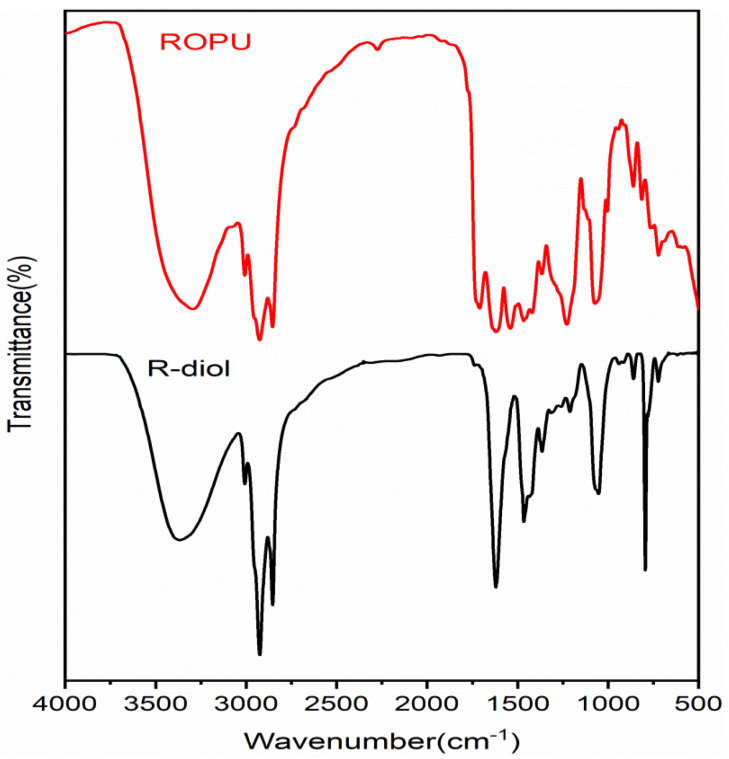
FT-IR spectra of R-diol and ROPU.

**Figure 2:**
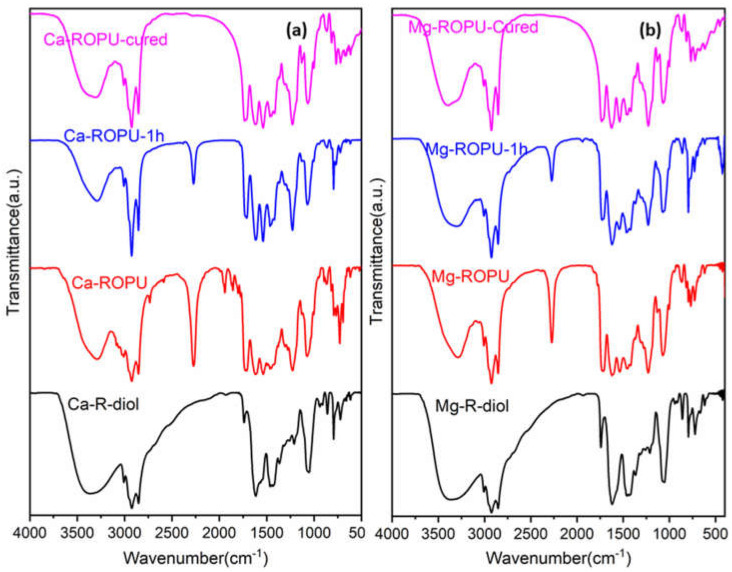
FT-IR spectra of Ca-R-diol, Ca-ROPU (**a**) and Mg-R-diol, Mg-ROPU (**b**).

FT-IR spectral technique was used to analyze the extent of reaction and curing of the two systems under study. The FT-IR spectra of R-diol, R-diol-PU, M-R-diol, and M-ROPU are given in Figure 1 and Figure 2. The distinctive vibrational peaks at 3365 cm^−1^ and 3008 cm^−1^ in the FTIR spectrum of R-diol (Figure 1) can be attributed to –OH and –C=C–, respectively. The FT-IR peaks at 2923–2854 cm^−1^ and 1615 cm^−1^, respectively, can be ascribed to –C–H stretching and (>C=O amide). Further evidence involving –CH bending vibrations was found at 1466 cm^−1^ and 1365 cm^−1^ [18]. Figure 1 represents the FT-IR spectrum of ROPU, which shows the presence of –OH/–NH groups with a vibrational peak at 3297 cm^−1^. Other peaks in R-diol-PU were very much similar, as observed in the spectrum of R-diol. However, some additional peaks were observed due to the reaction between R-diol and TDI. One small peak can be seen at 2274 cm^−1,^ which corresponds to the presence of some free –NCO groups in the polymeric chain. The peaks observed at 1619 cm^−1^ and 1536 cm^−1^ can be assigned to –C–C– stretching of the aromatic ring introduced through TDI and that of amide, respectively. The peak observed at 1710 cm^−1^ can be due to the –C=O group of urethanes. The FT-IR spectrum of R-diol-PU also reveals one peak at 1210–1152 cm^−1^ and another at 861–723 cm^−1^. These peaks can be attributed to stretching vibrations of (–C=O–O–C, –C–O–) and (Ar–C=C), respectively [21].

The FT-IR spectrum (Figure 2) of M-R-diol exhibits the most significant peak of the –OH group, which appears as broad absorption at 3365 cm^−1^. The characteristic peaks at 2923 cm^−1^ and 2853 cm^−1^ suggest the presence of the CH_2_ and CH_3_ group in the polymer chain, while the peak at 1739 cm^−1^ confirms the presence of conjugated carbonyl –C=O group of amide [22]. On the incorporation of TDI, the signal at 3367 cm^−1^ in the FT-IR spectra of M-ROPU (Figure 2) corresponding to the hydroxyl group gradually decreases, and the vibration peak shifts to the lesser stretching frequency area at 3288 cm^−1^, indicating the production of N–H bond in the M–ROPU. The appearance of a new peak at 2272 cm^−1^ confirms the presence of free –NCO, suggesting the successful reaction of TDI with M-R-diol [21]. The peaks at 1711 cm^−1^ can be assigned to C=O introduced through the soft segment, the peak at 1537 cm^−1^ can be correlated to NH–CO group, the peak at 1228 cm^−1^ corresponds to C-O group introduced from the hard segment, and finally, the peak at 1070 cm^−1^ represents C–C–O stretching vibration confirmed the establishment of urethane linkage and provided additional evidence that the M-ROPU was successfully formed. Further, the peaks observed at 1711 cm^−1^ can be attributed to the –C=O group of urethanes [23]. The FT-IR spectrum confirms the successful synthesis of R-diol, R-diol-PU, M-R-diol, and M-ROPU.

### 3.2. XPS Analysis

One of the most effective techniques for characterizing the surface chemistry of various materials is X-ray photoelectron spectroscopy (XPS). Through this method presence of various elements, their elemental compositions, electronic state, and chemical state are measured.

**Figure 3:**
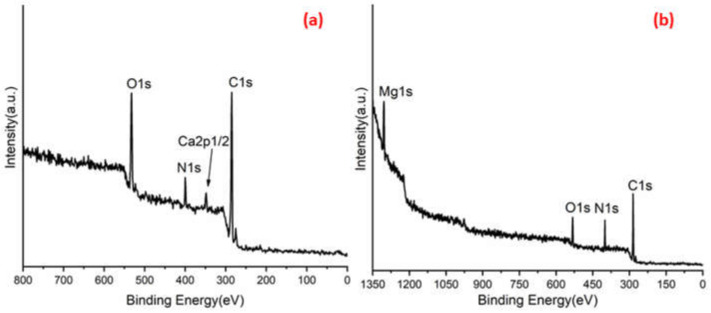
XPS spectra of (**a**) Ca-ROPU and (**b**) Mg-ROPU nanocomposite coatings.

**Figure 4:**
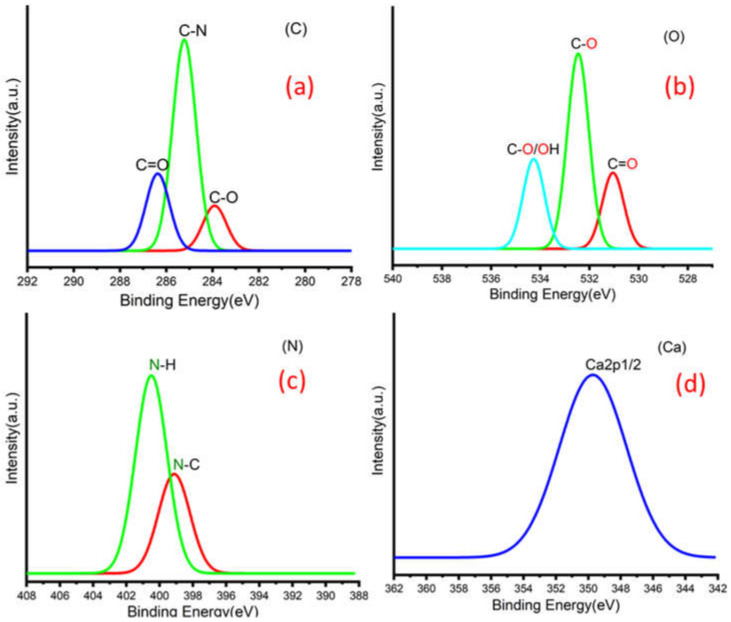
XPS spectra of deconvolution of Ca-ROPU nanocomposite (**a**) Carbon (**b**) Oxygen (**c**) Nitrogen and (**d**) Calcium.

**Figure 5:**
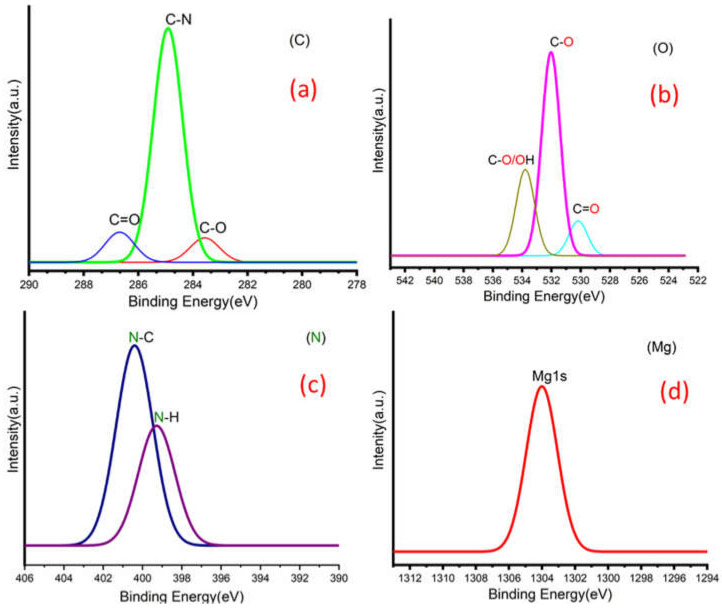
XPS spectra of deconvolution of Mg-ROPU nanocomposite (**a**) Carbon, (**b**) Oxygen, (**c**) Nitrogen, and (**d**) Magnesium.

Figure 3a,b shows C 1s peaks of Mg-ROPU/Ca-ROPU with three components according to its chemical structure. The central component at 284.93/285.21 eV was assigned to a carbon atom bonded to nitrogen (C–N). Other components at 283.59/283.88 eV and 286.70/286.38 eV were correlated to carbon singly bonded with oxygen (C–O) and doubly bonded with oxygen (C=O), respectively [24]. O 1s spectrum (Figure 4 and Figure 5) of Mg–ROPU/Ca-ROPU also shows three components. The dominant component at 533.78/534.27 eV corresponds to oxygen singly bonded with carbon. Other components located at 531.99/531.05 eV and 530.23/532.46 eV were attributed to C–O/OH and C=O groups, respectively [25]. N 1s spectrum (Figure 4 and Figure 5) of Mg-ROPU/Ca-ROPU shows only two components. The components found at 400.39/400.57 eV and 399.26/398.70 eV can be attributed to nitrogen bonded to hydrogen and nitrogen bonded to carbon, respectively [26]. This slight difference in binding energy values is slightly higher for Mg-ROPU, suggesting that the ROPU matrix is strongly coordinated to Mg ion as compared to Ca.

Further, Figure 5 shows Mg 1s spectrum located at 1303.90 eV corresponds to Mg 1s, which disbar the possibility of elemental Mg (1303.2–1303.8 eV) [27]. Similarly, in Figure 4 Ca 2p spectrum shows a peak centered at 349.71 eV, which can be attributed to Ca 2p1/2 electrons, which further eliminates the possibility of elemental Ca (345.9–346.6 eV) [28].

### 3.3. Curing of the Coatings

In order to analyze the curing/drying behavior of coating materials, the resin was placed over a Mild Steel Strip (MSS), and FT-IR spectra were collected at various intervals. FT-IR (Figure 2) was employed to monitor the variations in the –NCO group of PU’s absorption intensity. The curing of both systems was done at 120 °C for 1 h. At the beginning of the curing process, the FT-IR spectra of both freshly prepared Ca–ROPU/Mg-ROPU depict prominent peaks at 2274 cm^−1^ suggesting some free –NCO at the terminal positions. As the curing process proceeds further, this peak of –NCO can be seen to diminish, depicting the involvement of the –NCO group in the curing process. After the completion of the curing process, this peak completely disappears, confirming the complete consumption of the –NCO group, which results in a highly crosslinked structure [29].

In general, the free –NCO group present at the terminals of polymeric chains react with atmospheric moisture, followed by auto-oxidation of C=C groups, resulting in the drying/curing of M-ROPU coatings. The free –NCO groups react with ambient moisture to generate carbamic acid, which is extremely unstable. This quite unstable carbamic acid forms an amine-terminated polymer and emits CO_2_. In the presence of moisture, this amine-terminated polymer interacts with the NCO of TDI or free NCO of another M-ROPU moiety to create urea chains at room temperature. Because of the existence of double bonds, further curing occurs via crosslinking through auto-oxidation [30].

### 3.4. Morphological Studies

#### 3.4.1. XRD Analysis

**Figure 6:**
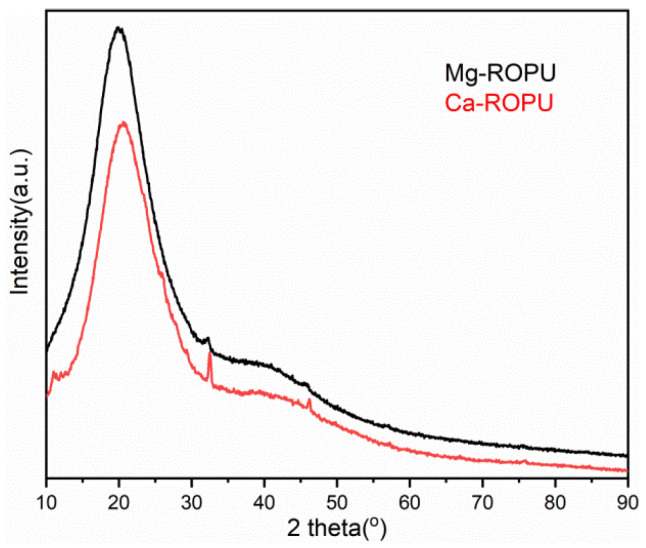
XRD of Ca-ROPU and Mg-ROPU nanocomposites.

X-Ray diffraction (Figure 6) was used to evaluate the diffraction pattern of Ca-ROPU and Mg-ROPU. Figure 6 shows the XRD pattern of polyurethane incorporated with calcium and magnesium. A broad peak centered at 2θ = 22° was observed for both the polymeric samples, which can be attributed to the characteristic diffraction pattern of PU. This peak appears to be a broad peak which may be due to the amorphous behavior of PU. Besides this, two small but sharp peaks with very low intensity were also observed in the case of Ca-ROPU and Mg-ROPU at 2θ = 33° and 2θ = 46°. These sharp peaks confirm that Ca and Mg have been impregnated in the PU matrix successfully [31].

#### 3.4.2. SEM Analysis

**Figure 7:**
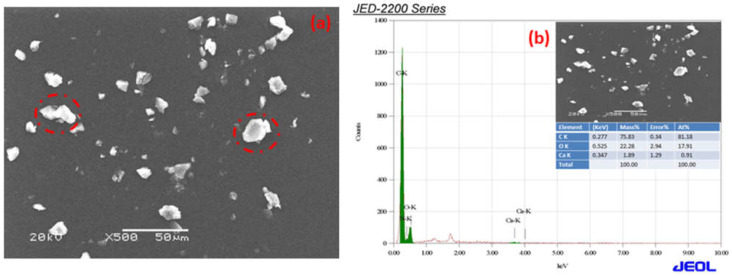
(**a**) SEM micrograph (round circle in the image represents Ca particles) and (**b**) EDS spectra of Ca-ROPU nanocomposite.

**Figure 8:**
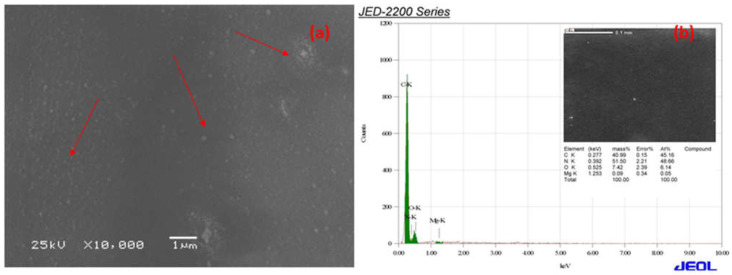
(**a**) SEM micrograph (Red color arrows indicating towards the Mg particles) and (**b**) EDS spectra of Mg-ROPU nanocomposite.

The SEM micrographs of Ca-ROPU and Mg-ROPU are shown in Figure 7a and Figure 8a, respectively. It is clear that even though the particles are evenly dispersed on the surface of the matrix, the distribution of Ca/Mg ions in the ROPU matrix results in the formulations having micro-Ca that has dispersed particles of different size, irregular shape, and agglomeration in some regions. Additionally, the particles in formulations containing nano-Ca/Mg are spherical, the granulometric distribution varies, and there is no sign of agglomeration because of the SEM range.

EDS was used to validate the chemical composition of the polymer coating in formulations, including Ca/Mg nanoparticles. Figure 7a,b illustrate the bright r spots are calcium and magnesium ions dispersed in the ROPU matrix [32].

#### 3.4.3. TEM

**Figure 9:**
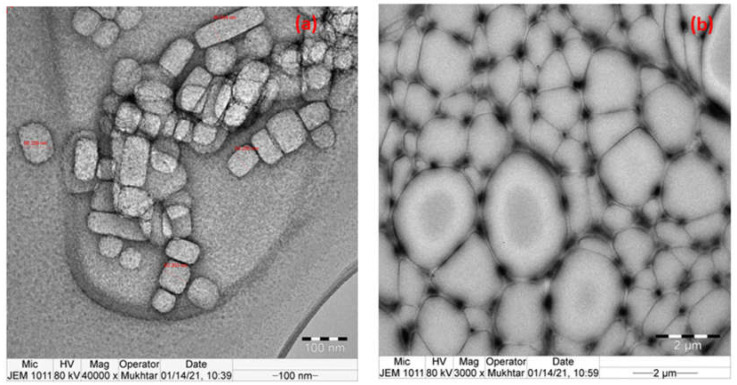
TEM image of (**a**) Ca-ROPU (100 nm), (**b**) Mg-ROPU nanocomposite (2 µm).

Figure 9a,b shows the results of TEM imaging that was carried out to further verify the dispersion of Ca/Mg ions within the ROPU matrix respectively. In Figure 9a, the bright section represents an organic component enclosing the dark inorganic component in a two-phase structure that is 58–63 nm in size and is dark owing to the presence of Ca ions and bright for ROPU. Whereas, in Figure 9b, a well-networked structure of the ROPU matrix can be seen surrounding the Mg ions.

The shape of the Ca/Mg nanoparticles could be clearly observed in the nanocomposite’s TEM picture, confirming the metal ions’ good dispersion inside the ROPU matrix. The grey ROPU matrix was evenly distributed with the nanoparticles (50–70 nm) without any buildup, as seen by the black spheres. The results confirm that a well-dispersed regular Ca-ROPU/Mg-ROPU nanocomposite has formed [33].

### 3.5. Thermal Analysis

**Figure 10:**
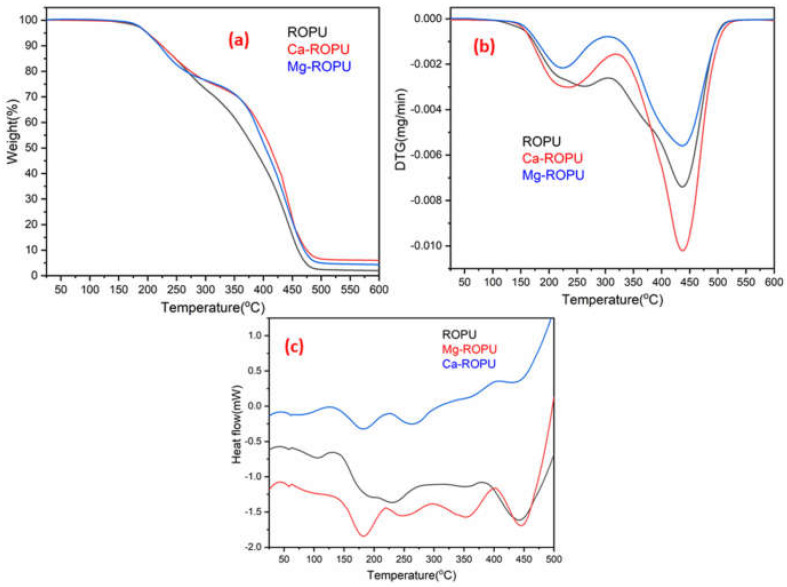
(**a**) TGA, (**b**) DTG, and (**c**) DSC thermograms showing thermal stability of ROPU, Ca-ROPU, and Mg-ROPU nanocomposite coatings.

Thermal analysis of the polymer samples was carried out using TGA, DSC, and DTG. The TGA (Figure 10a) of polymeric samples reveals that approx. 2% weight loss occurred at 175 °C, around 10% weight loss was seen at 225 °C. This weight loss can be due to adsorbed solvent and evaporation of the solvent. After this degradation step, the coating material degraded steeply up to 475 °C. This can be attributed to rupturing of alkyl chains, aromatic moieties, and urethane linkages [34]. In the case of other renewable resource-based polyesteramides, degradation generally starts around 150 °C. However, in this case, degradation starts at 175 °C, which can be attributed to the presence of urethane linkages and excellent crosslinking between the polymeric chains [35]. DTG thermogram (Figure 10b) of these coatings revealed the formation of two endothermic peaks. The first endothermic peak was observed at around 220 °C, and the second endothermic peak was observed at around 435 °C. These peaks further confirm the results of TGA.

DSC thermograms of RO and R-diol are shown in Figure S1. These thermograms clearly depict that the Tg value of RO lies near −20 °C, whereas that of R-diol lies around 13 °C. The DSC thermograms (Figure 10c) showed that the Tg value of ROPU, Ca/Mg-ROPU lie around 60 °C. Also, the formation of exotherms was seen at 130 °C, 225 °C, and 400 °C. The first exothermic peak in DSC appeared around 130 °C can be attributed to the release of entrapped solvent. The first exothermic peak that was observed around 225 °C only for Mg-ROPU and Ca-ROPU may be due to rupturing of weaker bonds and degradation of low molecular weight compounds such as urethane, aliphatic polymeric chains and ester linkages that exist in polymeric backbone [36]. Another endothermic peak observed around 350 °C in ROPU and Mg-ROPU, which was found to be slightly shifted and broadened in the case of Ca-ROPU, can be correlated to configurational changes since no weight loss is observed in this region [37]. On the other hand, a second exothermic peak which is observed at 400 °C, can be assigned to the breakdown of stronger bonds and degradation of high molecular weight compounds such as aromatic rings and conjugated double bonds [38].

### 3.6. Contact Angle Studies

**Figure 11:**
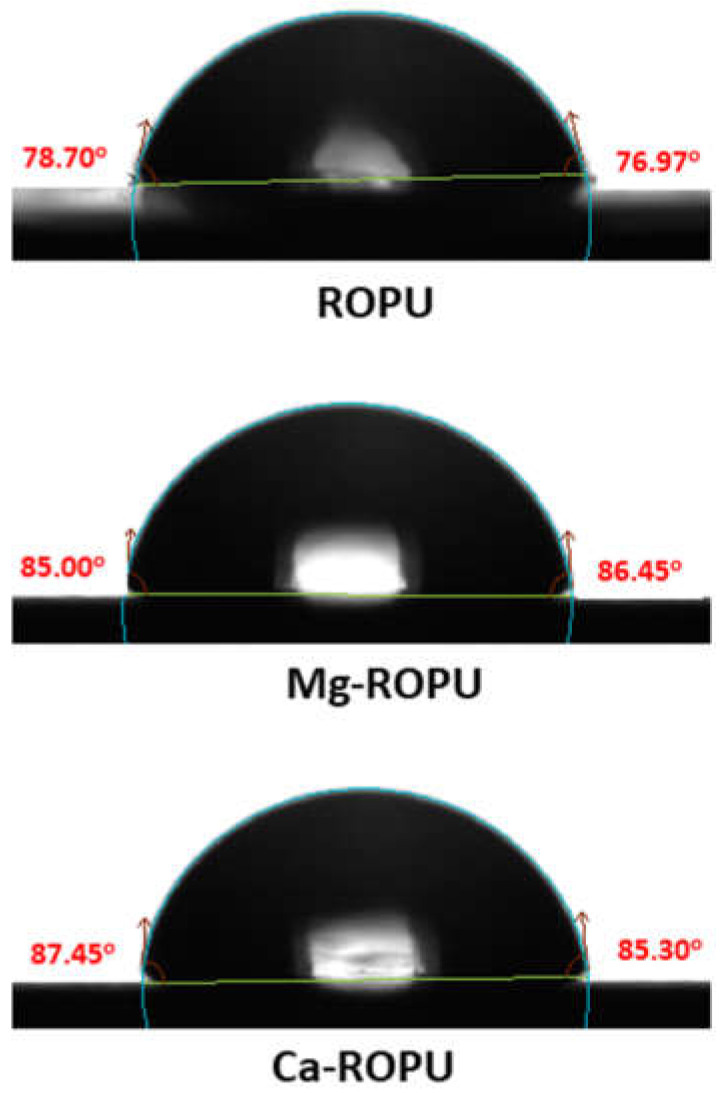
Contact angle images of ROPU, Mg-ROPU, and Ca-ROPU nanocomposite coatings.

Contact angle measurements (Figure 11) were carried out by the drop-casting method. 1 µL distilled water on the surface of plain ROPU and Ca/Mg-ROPU coatings to inspect the ability of water to maintain contact with a surface. Plain ROPU coatings revealed a contact angle value of 78° suggesting its hydrophilic nature. However, after impregnation of Ca and Mg, the contact angle values were observed to increase slightly. Ca-ROPU coatings acquired a contact angle value of 87° whereas, Mg-ROPU coatings exhibited a contact angle value of 85°. Thus, it can be concluded that impregnating ROPU with Ca and Mg increases the contact angle values, thereby decreasing the adsorption of water on the coating surface [39]. This lesser wettability of these coatings also suggests lower surface energy and good barrier performance towards water molecules which can lead to higher corrosion resistance performance of those coatings [40].

### 3.7. Physico Mechanical Properties

The thickness of these metallo-ROPU nanocomposite coatings was observed to be between 185–200 µm. The surface of these polymers was detected to be glossy with a gloss value (at 60 °C) of 100–105 G.U. The bend test demonstrated that all of the coatings were flexible, with no obvious surface ruptures or fractures. The metallo-ROPU nanocomposite coatings’ extraordinary flexibility is largely due to the polymeric backbone’s long fatty acid chain, which functions as an intrinsic plasticizer [41]. Furthermore, scratch hardness values enlarged from ROPU to Ca/Mg-ROPU, and were found to be maximum for Mg-ROPU, i.e., 3.4 kg, due to Ca/Mg ions electrostatically interacting with the polymer matrix and filling vacant space in the matrix [42]. Moreover, the homogeneously dispersed Ca/Mg ions behave as a restrictive obstruction that prevents the mobility of the tester’s tip, thereby increasing the scratch hardness values [43]. The cross-hatch tape test demonstrated that the coatings were firmly adhered to the metal surface, as after eradicating the tapes, zero squares were skinned off. The metallo-ROPU nanocomposite coating’s strong adhesive strength is principally owing to the locking action among metal ions and the ROPU matrix, which results in a 3D cross-linked structure [44]. The results of the cross-hatch tape test was found to be in good agreement with impact resistance tests. Table 1 shows all the physicomechanical results of ROPU, Ca-ROPU, and Mg-ROPU.

### 3.8. Anti-Corrosive Performance

#### 3.8.1. Potentiodynamic Polarization (PDP) Studies

**Figure 12:**
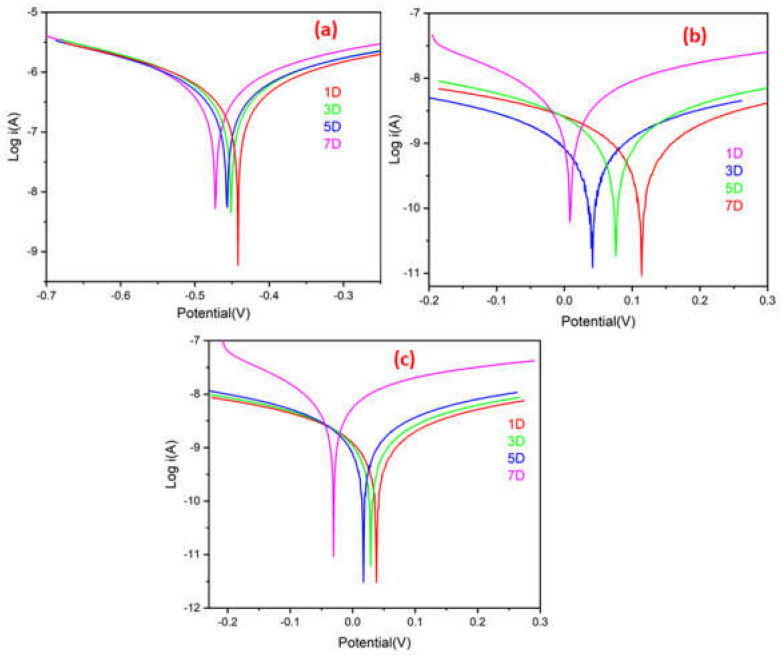
Tafel plot of (**a**) ROPU, (**b**) Ca-ROPU and (**c**) Mg-ROPU nanocomposite coatings.

A quantitative PDP experiment was used to evaluate the corrosion protection efficacy of ROPU and Ca/Mg-ROPU coatings for 7 days immersion time in a harsh corrosive environment using 3.5 wt. % NaCl. Figure 12 shows the polarization curves of samples coated with ROPU, Ca-ROPU, and Mg-ROPU in the specified environment, and Table 2 summarizes the data for their Tafel parameters, i.e., corrosion rate (CR), corrosion potential (E_corr_), and corrosion current density (i_corr_) [45].

E_corr_ and i_corr_ are essential for the evaluation of corrosion protection efficacy, and the corrosive disposition of metal as CR is inversely proportional to i_corr_ value and directly related to E_corr_ value [46]. So, the coating with lower i_corr_ and higher E_corr_ value offers enhanced corrosion protection. Plain ROPU showed an i_corr_ value of 3.063 × 10^−7^ A cm^−2^, confirming the substance’s good barrier characteristics.

ROPU showed an i_corr_ value of 3.063 × 10^−7^ A cm^−2^ after 7 days of immersion, confirming good barrier characteristics. On the other hand, for Ca-ROPU and Mg-ROPU coatings, i_corr_ values were observed to be 7.736 × 10^−10^ A cm^−2^ and 5.807 × 10^−9^ A cm^−2^, respectively. The above i_corr_ values are 2 and 3 order magnitude lesser than ROPU, which suggests that the incorporation of these metal ions further enhanced the corrosion resistance. Furthermore, the E_corr_ value of ROPU changed slightly, from −0.442 V to −0.472 V, whereas, E_corr_ values of Ca-ROPU, Mg-ROPU were determined between 0.114 V to 0.008 V and 0.037 V to −0.030 V respectively, It indicates that E_corr_ values of Ca-ROPU and Mg ROPU are greater than ROPU after 7 days immersion. This confirms the addition of Ca/Mg ions increases the corrosion resistance capability of ROPU coating. 

Ca-ROPU and Mg-ROPU coatings exhibit significantly stronger water-repellent behavior, good mechanical properties, and a large specific surface area in the ROPU matrix, resulting in significantly higher E_corr_ and lower i_corr_ values than ROPU coatings [47]. Additionally, the corrosion rates and polarization resistance values of these coatings are in close agreement with the respective corrosion current densities of the coatings made of ROPU and metallo-ROPU nanocomposites. The higher E_corr_ value of Mg-ROPU coatings is observed to be about 0.008 V even after 7 days, corresponding to the development of a protective passive oxide layer.

The overall PDP results show that Mg-ROPU is more efficient with higher E_corr_ and lower i_corr_ corrosion resistance than Ca-ROPU. These experiments suggest that metallo-ROPU nanocomposites are strong and long-lasting. These coatings exhibit excellent anti-corrosion performance, which is due to their high physical barrier and adhesion property, thereby preventing corrosive ions from penetrating the coating surface.

#### 3.8.2. Electrochemical Impedance Spectroscopy

**Figure 13:**
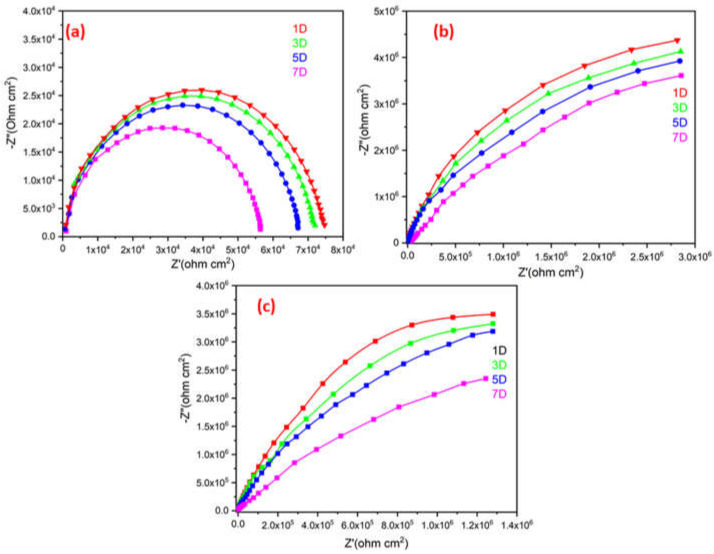
Nyquist plot of (**a**) ROPU, (**b**) Ca-ROPU, and (**c**) Mg-ROPU nanocomposite coatings.

Figure 13 illustrates a single semicircle on a Nyquist plot for the plain ROPU, and Ca-ROPU/Mg-ROPU acquired after exposing it to 3.5 wt. % sodium chloride (NaCl) solution for 7 days. All the EIS parameters are tabulated in Table 3. The ROPU Nyquist plot initially (1st day) comprised a single semicircle at low and high frequencies. The Nyquist plots then exhibit the same trend for the following seven days, with a semicircle at high frequency, which indicates the solution resistance of these coatings. This result suggests that after 5 days of immersion, the chloride ions slowly permeate into the coating through tiny pores. Additionally, as immersion duration increases, the radius of impedance decreases.

The Nyquist plots for Ca-ROPU/Mg-ROPU coating also revealed only one semicircle for the 7 days, with the decrease in radius of the semicircle with the increase in immersion time. This indicates that the material is entirely capacitive and has a high barrier effect. Due to the homogeneous dispersion of metal ions inside the ROPU matrix functioning as a second compact physical barrier, the Nyquist plot of coating demonstrates a linear connection with the real and imaginary impedance portions [48]. With increasing immersion time, there was a reduction in the capacitive loop. The long-term anti-corrosive effectiveness of these coatings (after 7 days) is attributed to the successful impregnation of Ca/Mg into ROPU, locking the pathways, giving additional complication and tortuousness for ion diffusion, causing the prevent in penetration of chloride ions [49]. The EIS data of these coatings were found to be in good agreement with the PDP results discussed earlier. Overall, Mg-ROPU showed the best results among all in both PDP and EIS tests.

**Figure 14:**
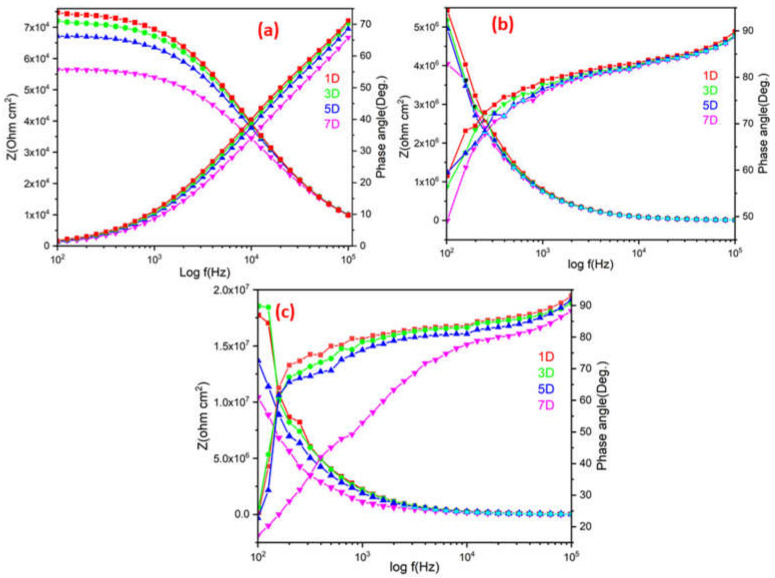
Bode-theta plot of (**a**) ROPU, (**b**) Ca-ROPU, and (**c**) Mg-ROPU nanocomposite coatings.

Figure 14 shows the Bode diagrams derived from the impedance|Z|measurement. It provides information on impedance at low and high frequencies during coating water uptake, which indicates coating resistance and capacitance, respectively. In general, the high frequency (i.e., 10^2^ to 10^5^ Hz) curve of the Bode plot corresponds to the coating protection. Figure 14 also demonstrates that, as compared to plain ROPU, the modulus at low frequency for ROPU and Ca-ROPU/Mg-ROPU coatings is significantly high. This is due to the ability of the coatings to lock the current between the anodic and cathodic zones. With increasing immersion duration, the impedance values for all coated substrates drop in the series ROPU > Ca-ROPU > Mg-ROPU, which indicates the restrictive behavior of these coatings, which increases due to the impregnation of metal ions into the polymer backbone. The impedance value of Ca-ROPU was observed to drop up to 4.0 × 10^6^ Ω cm^2^ after 7 days of immersion. However, Mg-ROPU remained at 1.0 × 10^7^ Ω cm^2^ after the same days, which is significantly greater than Ca-ROPU.

Furthermore, the phase angle plot was used to predict the characteristic of anti-corrosive coatings as it determines damage or delamination occurring at the time of immersion. The phase angle plots of Ca-ROPU/Mg-ROPU revealed only one time constant and a high phase angle of >90° for Mg-ROPU and 90° for Ca-ROPU throughout the immersion duration. The angle of Ca-ROPU/Mg-ROPU coating reached about 90° with no breakpoint frequency, indicating undamaged coatings until the end of the experiment in both cases.

## 4. Conclusions

In this study, metallo-ROPU nanocomposite coatings were effectively created for the anti-corrosive application. The structure of ROPU and metallo-ROPU were characterized by FT-IR. The morphology of these materials was checked by TEM and SEM analysis. Their thermal stability was determined by TGA thermograms. The coating significantly improved the physicomechanical characteristics. PDP and EIS methods were applied to estimate the corrosion performance of coated for 7 days in a 3.5% NaCl solution. Mg-ROPU coating represented a very low corrosion rate of 8.342 × 10^−5^ mpy, high impedance modulus value of 1.0 × 10^7^ Ω cm^2^, and least corrosion current density, i.e., 7.73 × 10^−10^ A cm^−2^ and therefore were regarded as best. The establishment of a strong secondary barrier (offering dual protection) due to Ca/Mg impregnation leads to the enhancement of the corrosion resistance performance of ROPU nanocomposites. The results of these experiments demonstrated that the suggested bio-based metallo-ROPU coatings have a probable opportunity to be applied in coating industries requiring better corrosion inhibition performance.

## Figures and Tables

**Table 1:** Physicomechanical properties of ROPU, Ca-ROPU, and Mg-ROPU.

Tests	ROPU	Ca-ROPU	Mg-ROPU
Scratch hardness (kg)	2.5	3.0	3.4
Impact (lb/inch)	100 Pass	120 Pass	150 Pass
Bending (1/8″)	Pass	Pass	Pass
Pencil hardness	3H	3H	4H
Cross Hatch (%)	95	95	100
Gloss at 60°	70	100	105
Dry-to-touch time (min)	5	5	3–4
Thickness (micron)	200	185	130

**Table 2:** The Tafel parameters for ROPU, Ca-ROPU, and Mg-ROPU coating materials were under 3.5% NaCl solution for a period of 7 days.

Immersion Time (Day)	E_corr_ (V)	i_corr_ (A/cm^2^)	Corrosion Rate (mm/Year)	LPR (Ω)	OCP(V)
ROPU					
1	−0.442	2.019 × 10^−7^	2.345 × 10^−3^	1.314 × 10^5^	−0.427
3	−0.451	2.833 × 10^−7^	3.346 × 10^−3^	1.178 × 10^5^	−0.432
5	−0.457	2.836 × 10^−7^	3.396 × 10^−3^	1.121 × 10^5^	−0.438
7	−0.472	3.063 × 10^−7^	3.559 × 10^−3^	1.035 × 10^5^	−0.464
Ca-ROPU					
1	0.114	6.841 × 10^−10^	6.949 × 10^−5^	6.312 × 10^7^	0.064
3	0.075	7.168 × 10^−10^	7.358 × 10^−5^	5.336 × 10^7^	0.064
5	0.040	7.348 × 10^−10^	7.539 × 10^−5^	4.936 × 10^7^	0.011
7	0.008	7.736 × 10^−10^	8.342 × 10^−5^	1.278 × 10^7^	0.054
Mg-ROPU					
1	0.037	1.154 × 10^−9^	1.342 × 10^−5^	4.382 × 10^7^	0.023
3	0.028	1.279 × 10^−9^	1.487 × 10^−5^	3.846 × 10^7^	0.017
5	0.017	1.394 × 10^−9^	1.620 × 10^−5^	3.153 × 10^7^	0.013
7	−0.030	5.807 × 10^−9^	6.748 × 10^−5^	7.093 × 10^6^	0.041

**Table 3:** The electrochemical impedance (EIS) parameter for ROPU, Ca-ROPU, and Mg-ROPU coating materials under 3.5 wt. % NaCl solution for a period of 7 days.

ROPU				
Immersion Time	R_s_ (Ω)	R_ct_ (KΩ)	Capacitance (pF)	χ^2^
1	121	8.45	182	0.950
3	136	7.30	189	1.036
5	142	6.35	194	1.173
7	143	6.06	198	1.560
Ca-ROPU				
1	138	15.3	64.9	0.610
3	148	11.7	63.3	0.391
5	279	11.11	74.4	1.133
7	421	5.32	80.0	1.475
Mg-ROPU				
1	427	66.5	242	1.59
3	424	63.7	263	1.51
5	447	59.6	265	0.82
7	420	46.3	260	0.59

## Data Availability

Data contained within the Article and Supplementary File.

## Supplementary Materials

The following supporting information can be downloaded at: https://www.mdpi.com/article/10.3390/ma15238374/s1, Figure S1: DSC thermogram of RO and R-diol.
