# Supplementary material for: Development of Metallo (Calcium/Magnesium) Polyurethane Nanocomposites for Anti-Corrosive Applications
Source: Materials (Basel). 2022 Nov 24;15(23):8374. doi: 10.3390/ma15238374 (PMC9738934; doi:10.3390/ma15238374)
Supplement: Supplementary file 1 [file materials-15-08374-s001.zip › materials-1982309-supplementary.pdf]

Development of metallo (calcium/magnesium) polyurethane nanocomposites for anti-corrosive applications

Manawwer Alam, Mohammad Altaf, Mukhtar Ahmed, Mohammed Rafi Shaik, Rizwan Wahab, Jilani Purusottapatnam Shaik , Mohammad Shahzad Samdani and Ashfaq Ahmad

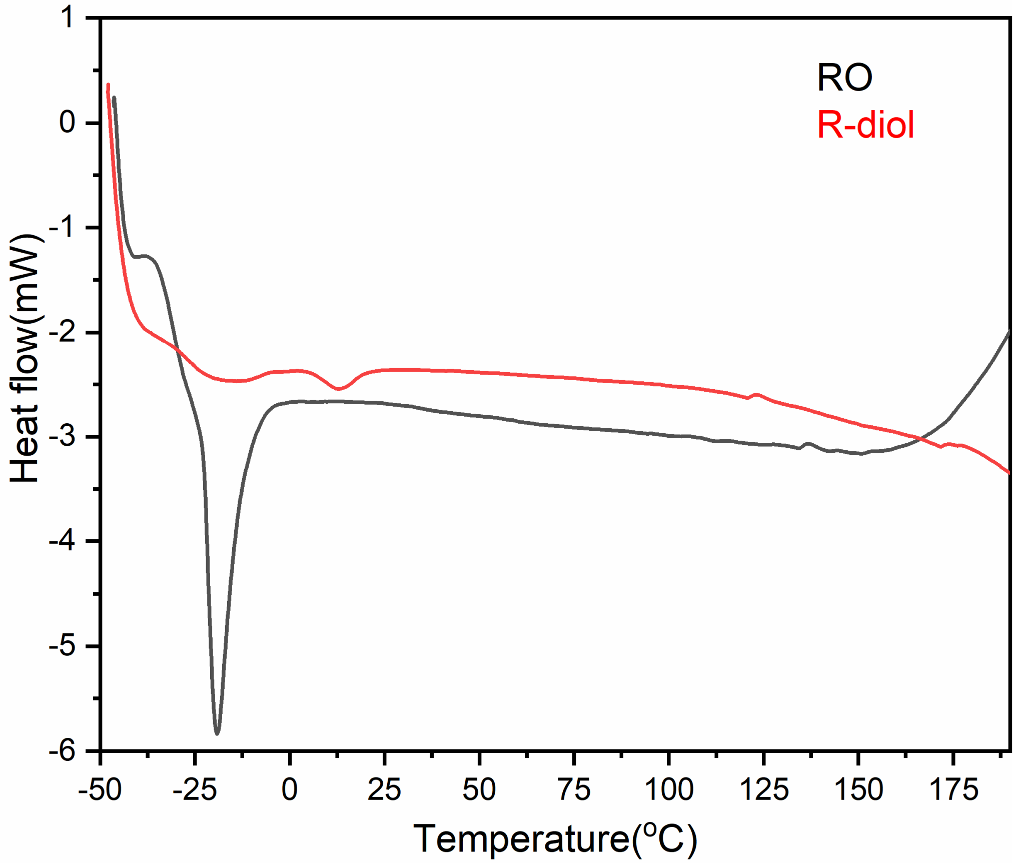

Figure S1: DSC thermogram of RO and R-diol
